# Prediction of pneumoconiosis by serum and urinary biomarkers in workers exposed to asbestos-contaminated minerals

**DOI:** 10.1371/journal.pone.0214808

**Published:** 2019-04-04

**Authors:** Hsiao-Yu Yang

**Affiliations:** 1 Institute of Environmental and Occupational Health Sciences, National Taiwan University College of Public Health, Taipei, Taiwan; 2 Department of Public Health, National Taiwan University College of Public Health, Taipei, Taiwan; 3 Department of Environmental and Occupational Medicine, National Taiwan University Hospital, Taipei, Taiwan; University of Cincinnati College of Medicine, UNITED STATES

## Abstract

Workers processing nephrite, antigorite, or talc may be exposed to paragenetic asbestos minerals. An effective screening method for pneumoconiosis in workers exposed to asbestos-contaminated minerals is still lacking. The objective of this study was to assess the diagnostic accuracy of serum and urinary biomarkers for pneumoconiosis in workers exposed to asbestos-contaminated minerals. We conducted a case-control study in a cohort of stone craft workers in Hualien, where asbestos, nephrite, antigorite, and talc are produced. A total of 140 subjects were screened between March 2013 and July 2014. All subjects received a questionnaire survey and a health examination that included a physical examination; chest X-ray; and tests for standard pulmonary function, fractional exhaled nitric oxide, serum soluble mesothelin-related peptide (SMRP), fibulin-3, carcinoembryonic antigen (CEA), and urinary 8-Oxo-2'-deoxyguanosine (8-OHdG)/creatinine. After excluding subjects with uraemia and chronic obstructive pulmonary disease (COPD), we included 48 subjects with pneumoconiosis and 90 control subjects without pneumoconiosis for analysis. In terms of occupational history, 43/48 (90%) case subjects and 68% (61/90) of the control subjects had processed asbestos-contaminated minerals, including nephrite, antigorite, and talc. The case group had decreased pulmonary function in forced vital capacity (FVC), forced expiratory volume in one second, and forced expiratory flow between 25% and 75% of the FVC. The levels of SMRP, fibulin-3, urinary 8-OHdG/creatinine, and CEA were higher in the case group than in the control group. Subjects exposed to nephrite had significantly higher SMRP levels (0.84 ± 0.52 nM) than subjects exposed to other types of minerals (0.60 ± 0.30 nM). A dose-response relationship was observed between the SMRP level and the severity of pneumoconiosis. Machine learning algorithms, including variables of sex, age, SMRP, fibulin-3, CEA, and 8-OHdG/creatinine, can predict pneumoconiosis with high accuracy. The areas under the receiver operating characteristic curves ranged from 0.7 to 1.0. We suggest that SMRP and fibulin-3 could be used as biomarkers of pneumoconiosis in workers exposed to asbestos-contaminated minerals.

## Introduction

Pneumoconiosis is an important occupational lung disease caused primarily by inhalation of mineral dust from asbestos and crystalline silica. Inhalation of asbestos causes an estimated 107,000 deaths per year worldwide [1, 2] and has been gradually banned in many countries. However, some asbestos minerals, such as amphibole and serpentine minerals, can exist in compacted masses and are not regulated as asbestos; therefore, they are still widely used as building materials, decorations, and jewellery [3, 4]. Recent studies have shown that workers processing nephrite jade, a non-asbestiform tremolite-actinolite asbestos mineral, can release asbestiform fibre, increasing the risk of pneumoconiosis and lung cancer [5]. When workers process serpentinite rocks, such as antigorite or talc, workers may also be exposed to paragenetic asbestos minerals [6, 7].

Hualien is in the convergent plate-boundary zone and is rich in metamorphic rocks and serpentinite [8]. Serpentinite rock consists of mainly serpentine minerals (i.e., chrysotile and antigorite) and small amounts of other minerals, such as talc and tremolite-actinolite [9]. Fengtian is the main production area for nephrite, antigorite, and talc in Hualien. Chrysotile, tremolite, and actinolite asbestos were also produced in Fengtian between 1938 and 1985 [10]. When workers process nephrite, antigorite, or talc, which could contain paragenetic asbestos, they are exposed to non-asbestiform and a proportion of asbestiform elongated mineral particles (EMPs) and have an increased risk of cancer [7, 11]. Similar exposures to both non-asbestiform and asbestiform EMPs in occupational settings have been reported among taconite miners in Minnesota [12] and talc miners in upstate New York [13]. These workers are exposed to both non-asbestiform and asbestiform EMPs in the occupational setting, but they are not defined as traditional asbestos workers. The occupational hazards remain unclear, and no regulations exist regarding the mixed asbestiform and non-asbestiform EMP exposure environment [3].

Some biomarkers have been used to screen asbestos-related diseases. Soluble mesothelin-related peptide (SMRP) is currently the most common biomarker for mesothelioma and can also be used as an indicator of asbestos exposure [14, 15]. A combination measurement of serum SMRP and carcinoembryonic antigen (CEA) could improve the accuracy of the detection of asbestos-related diseases [16]. Fibulin-3 is a diagnostic marker for mesothelioma [17]. Urinary 8-hydroxy-2’-deoxyguanosine (8-OHdG) has been used to measure the oxidative stress caused by asbestos and quartz [18]. The objective of this study was to assess the diagnostic accuracy using the serum and urinary biomarkers for pneumoconiosis in workers exposed to asbestos-contaminated minerals.

## Materials and methods

### Study subjects

We recruited study subjects from a health surveillance for stone workers in Hualien between March 2013 and July 2014. These stone workers processed jade artefacts, building materials, decorations, sculptures, vases, or urns. The study was approved by the Research Ethics Committee of Hualien Tzu-Chi Hospital (No. IRB103-31-B). All participants provided written informed consent before enrolment in the study.

### Medical examinations and questionnaire survey

We arranged a health examination for the study subjects. We conducted face-to-face interviews to confirm their occupational history of stone working, including the years they started and ended, the number of years they had accumulated for these types of stones, and the tasks in which they were involved. The questionnaire was developed based on more than 200 filed surveys that were conducted by occupational physicians and industrial hygienists, and it was pretested using senior stone workers in Hualien to correct any ambiguous wording [5]. Cigarette smoking history was obtained using the standard ATS-DLD-78-A questionnaire, which included cigarette-smoking history. All subjects received a health examination that included a physical examination; chest X-ray (computed radiography FCR XG5000, Fuji Photo Film, Tokyo, Japan); standard pulmonary function test; fractional exhaled nitric oxide (FeNO) test; blood tests, including complete blood count, serum creatinine, aspartate aminotransferase, and alanine aminotransferase; urinalysis; and tests for serum SMRP, fibulin-3, CEA, and urinary 8-OHdG. We collected blood and urine samples after overnight fasting. The measurement of FeNO followed the ARS/ERS recommendation [19]. The physician performed a physical examination and inquired about symptoms and signs. The results of the physical examination were recorded point-by-point on a structured record to exclude any cardiac or major systemic diseases that may cause shortness of breath, including asthma, anaemia, heart disease, and thyroid disease. During the physical examination, the physician asked the workers whether they had experienced chest pain or shortness of breath, and participants were evaluated for clubbing of fingers. An occupational physician used a semi-structured questionnaire to review the subject’s medical history, including the occurrence of bronchiectasis, pleurisy/pleuritis, tuberculosis, chronic obstructive pulmonary disease (COPD), allergic rhinitis, sinusitis, scleroderma, systemic lupus erythaematosus, rheumatoid arthritis, and cancer. To prevent misunderstanding of medical terms, we used the ATS-DLD-78-A questionnaire to obtain information about the symptoms of lung disease and the individual and family histories of lung disease. The diagnoses of diseases were ascertained by medical doctors and were then confirmed using the subject’s medical records. To assess the severity of pulmonary fibrosis and biomarkers, we excluded subjects with other diseases that can cause pulmonary fibrosis rather than pneumoconiosis. Two physicians read chest X-rays according to the International Labour Office (ILO)/International Classification of Radiographs of Pneumoconiosis (ICRP). The readers were blinded (masked) to the results of the other tests and the clinical information. Physicians read chest X-rays by comparing them with ILO/ICRP 2000 standardized films and recorded the findings in the standard roentgenographic interpretation format of the NIOSH Coal Workers’ Health Surveillance Program. The exclusion criteria for the study subjects included a medical history of pulmonary tuberculosis, COPD diagnosed by chest physicians that required regular medication, a medical history of autoimmune diseases, an acute infection (defined as a white blood cell count greater than 10.0 x 10^3^/μL), and uraemia (defined as an estimated glomerular filtration rate less than 60 mL/min with uremic symptoms or were receiving dialysis treatment).

### Diagnosis of pneumoconiosis

Pneumoconiosis is a lung disease resulting from mineral dust deposition in the lung and the subsequent host response. Since the inhalation of a wide variety of types of mineral dust can result in pneumoconiosis, the diagnostic criteria differ for different minerals. However, three major criteria are usually required for the diagnosis of pneumoconiosis. The first criterion is sufficient exposure to mineral dust known to cause pneumoconiosis with an appropriate latency period. Exposures to silica, coal or asbestos occur most commonly in an occupational setting. The second criterion is the recognition of a characteristic chest radiograph, which meets published standards for the diagnosis of pneumoconiosis. Although respiratory symptoms and impairment in lung function commonly occur in workers diagnosed to have pneumoconiosis, neither is requisite for the diagnosis. The third criterion is the absence of an illness that might mimic pneumoconiosis [20]. This study used the clinical diagnosis of pneumoconiosis by experienced occupational physicians based on medical history, physical examination, occupational history of long-term exposure to mineral dust, and parenchymal abnormalities consistent with pneumoconiosis with a profusion score ≥ 1/0.

### Collection and measurement of biomarkers

All samples were collected and then analysed in an ISO15189-accredited medical laboratory. Blood was collected in a serum-separating tube, gently inverted at least 5–8 times and allowed to clot in a vertical position for at least 30 minutes at ambient temperature. The blood was then centrifuged at 1,800 x g for 10 minutes at 4°C within one hour after sampling. After centrifugation, serum was aliquoted and stored at -20°C for the fibulin-3 test and at -70°C for the SMRP test. We collected 10 mL of urine in a sterile container and divided it into aliquots of 1 mL per vial, which were stored at -70°C until the 8-OHdG analysis was performed. The serum SMRP level was analysed with a sandwich enzyme-linked immunosorbent assay (ELISA) (MESOMARK, Fujirebio Diagnostics, Malvern, PA, USA), and the absorbance was read at 405 nm using an ELISA plate reader (BioTek Instruments Inc., Winooski, VT, USA) [21]. The concentrations of SMRP were then extrapolated from the six-point standard curve (0–32 nM) and are expressed in nM. The fibulin-3 level in the serum was measured using a human fibulin-3 ELISA kit (Cloud-Clone Corp, Houston, Texas, USA). Urinary 8-OHdG was analysed using a competitive ELISA kit (E0660Ge, EIAab Science, Wuhan, P.R. China). All ELISA analyses followed the manufacturer’s instructions and were measured in duplicate. Because hydration status may influence the concentration of urinary 8-OHdG [22], the concentration of 8-OHdG was expressed relative to the urinary creatinine level (8-OHdG/creatinine). Samples were coded, and research personnel were blinded to the clinical information.

### Statistics

We used logistic regression to calculate the area under the receiver operating characteristic curve (AUROC) for each variable. Those who had an AUROC value greater than 0.5 were then selected to construct a prediction model for pneumoconiosis. We applied six machine learning methods of decision tree, extreme gradient boosting [23], random forests [24], support vector machines [25], generalized linear models, and neural networks [26] to build the prediction models. Using the clinical diagnosis of pneumoconiosis as the reference standard, we assessed the prediction accuracy by the AUROC of the models. Statistical calculations were performed using SAS 9.4 software (SAS Institute Inc., Cary, NC, USA) and the R statistical language using the rattle package [27]. The statistical analysis protocol can be accessed at http://dx.doi.org/10.17504/protocols.io.x8dfrs6.

## Results

**Fig 1:**
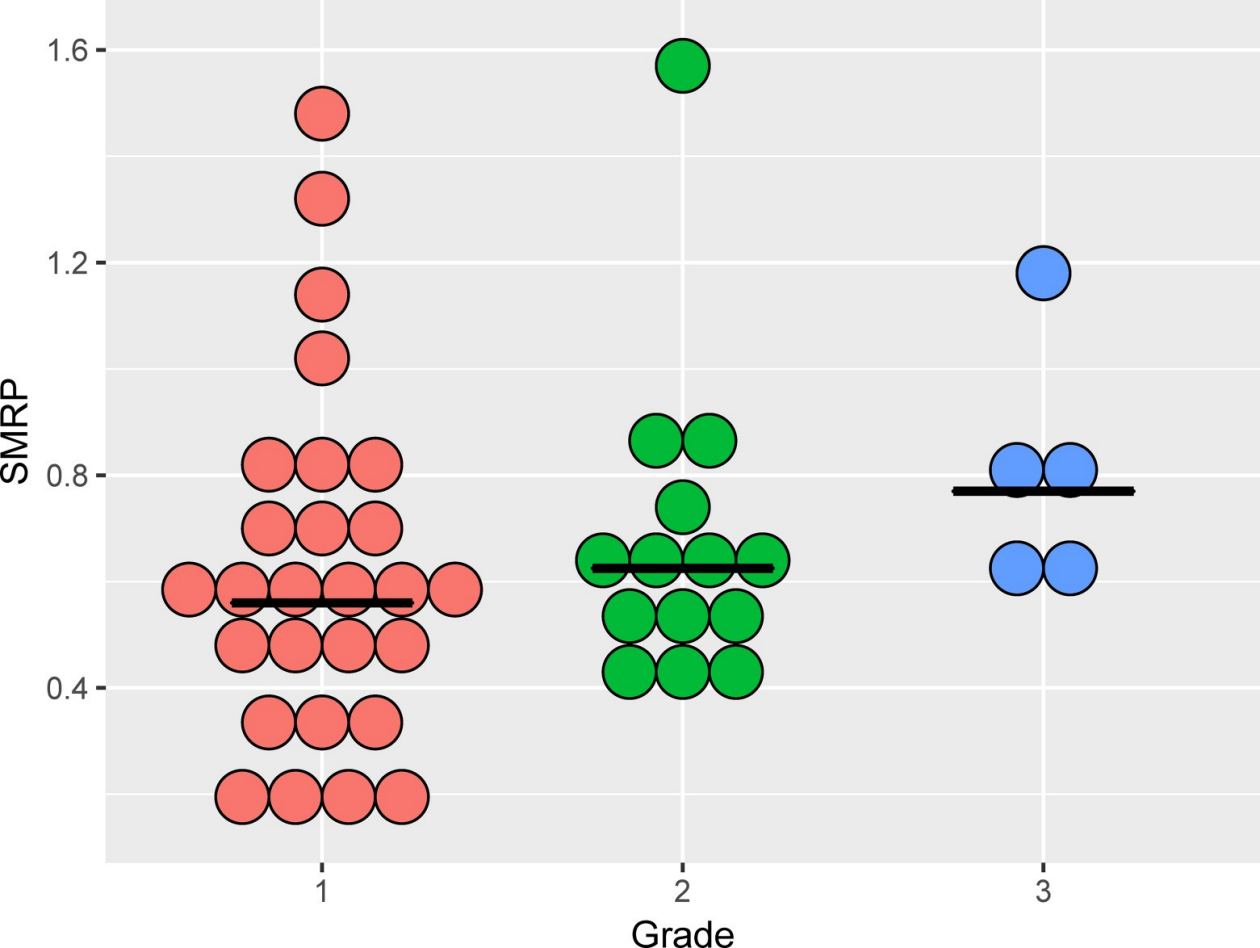
Scatter dot plot showing SMRP levels in subjects with pneumoconiosis with different severity. The horizontal bars indicate the medians. A dose-response relationship was observed between the SMRP levels and the severity of pneumoconiosis. Grade 1: Profusion ≥ 1/0, no pulmonary function abnormality, and no audible crackles on physical examination. Grade 2: Profusion ≥ 1/0, the presence of restrictive type pulmonary function abnormality, and no audible crackles on physical examination. Grade 3: Profusion ≥ 1/0, the presence of restrictive type pulmonary function abnormality, and the presence of audible crackles on physical examination.

**Fig 2:**
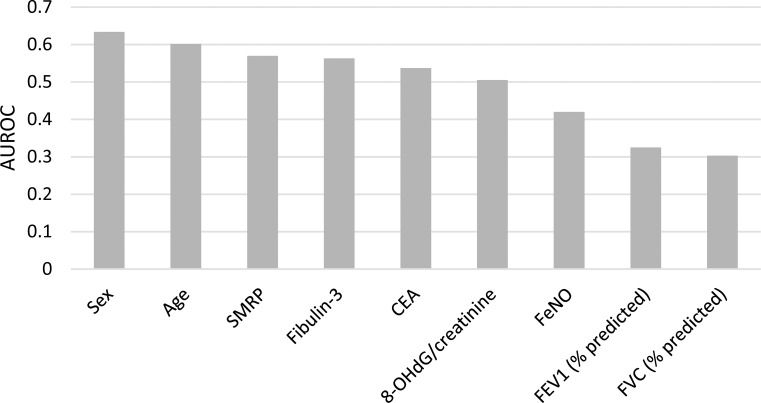
The AUROC for pneumoconiosis. The AUROCs of sex, age, SMRP, fibulin-3, CEA, and 8-OHdG/creatinine were greater than 0.5, suggesting that their predictive capacities perform better than random. A single breath test for FeNO, FEV1, or FVC cannot predict pneumoconiosis.

**Fig 3:**
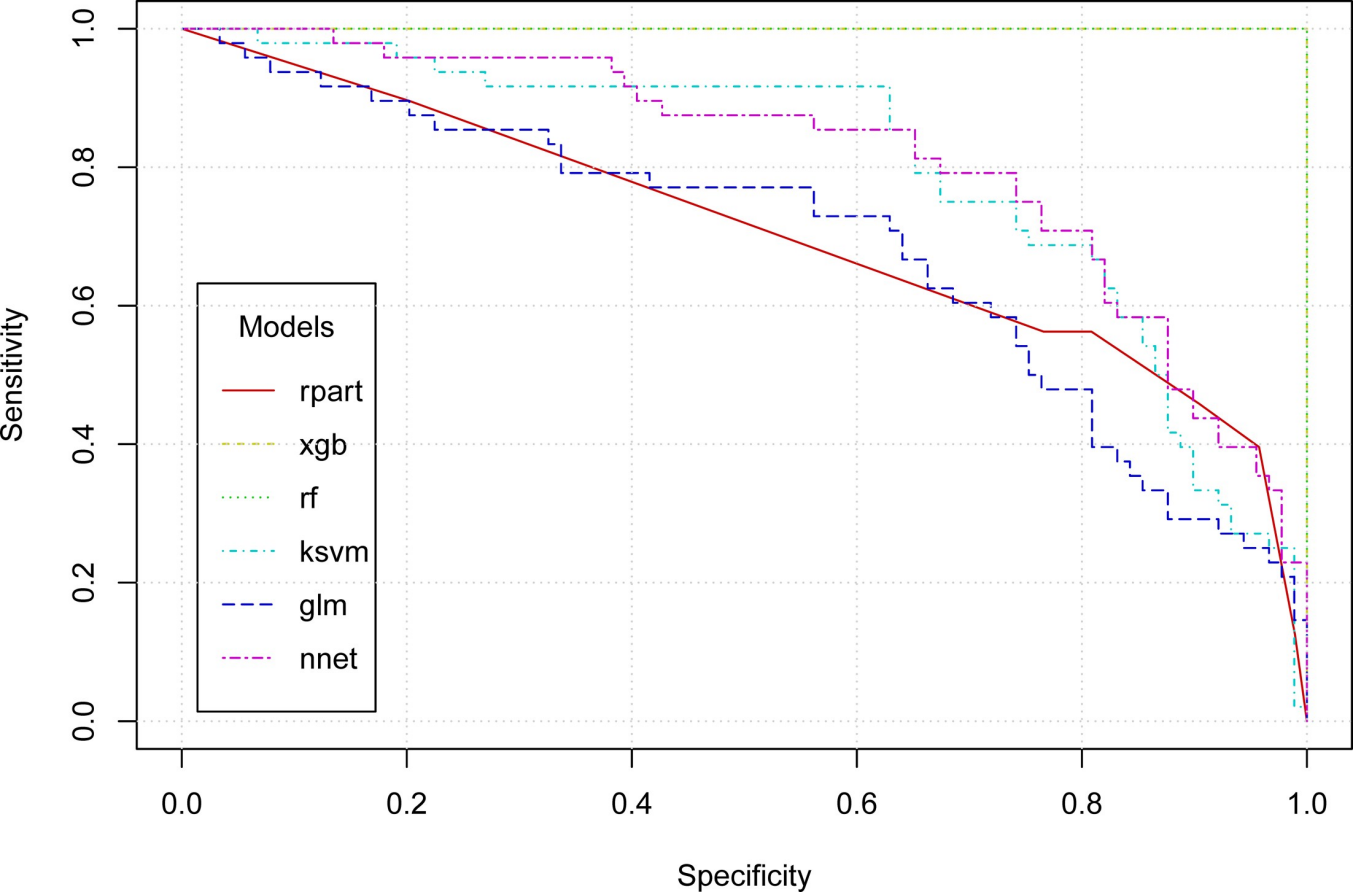
Receiver operating characteristic curves for pneumoconiosis predicted by six machine learning algorithms. We included the prediction variables of age, sex, SMRP, fibulin-3, CEA, and 8-OHdG/creatinine in the model and used six machine learning algorithms to establish a prediction algorithm. Machine learning algorithms use R packages of the decision tree (rpart), extreme gradient boosting (xgb); random forests (rf), support vector machines (ksvm), generalized linear models (glm), and neural networks (nnet). The AUROCs ranged from 0.7 to 1.0.

A total of 140 subjects were screened between March 2013 and July 2014. There were no cases of malignant pleural mesothelioma (MPM). After excluding one subject with uraemia and one subject with COPD, a total of 138 subjects satisfied the inclusion criteria, including 48 subjects with pneumoconiosis and 90 control subjects without pneumoconiosis. In the case group, 32 subjects had primarily small (width 1.5–10 mm) irregular opacities in the chest X-ray, 13 subjects had primarily small round opacities, and three had mixed forms. The case group had decreased pulmonary function in forced vital capacity (FVC), forced expiratory volume in one second (FEV1), and forced expiratory flow between 25% and 75% of the FVC (FEF25-75). The levels of SMRP, fibulin-3, urinary 8-OHdG/creatinine, and CEA were higher in the case group than in the control group (Table 1). In occupational history, 43/48 (90%) case subjects and 68% (61/90) of the control subjects had processed asbestos-contaminated minerals, including nephrite, antigorite, and talc. The mean SMRP values were 0.84 ± 0.52 nM in workers exposed to nephrite and 0.60 ± 0.30 nM in workers exposed to other minerals (*p*-value of one-tailed Wilcoxon rank sum test = 0.04). To explore the relationship between the severity of pneumoconiosis and the SMRP level, we categorized pneumoconiosis into three grades based on the profusion in the chest X-ray, the presence of a restrictive type of pulmonary function impairment, and audible crackle, which is an important clinical sign of pneumoconiosis in a physical examination [28]. A dose-response relationship was observed between the SMRP level and the severity of pneumoconiosis (Fig 1). Among individual biomarkers, SMRP had the highest AUROC, followed by fibulin-3, CEA, and 8-OHdB (Fig 2). Machine learning algorithms composed of sex, age, SMRP, fibulin-3, CEA, and 8-OHdB can predict pneumoconiosis with high accuracy. The AUROCs of different machine learning algorithms ranged from 0.7 to 1.0 (Fig 3).

**Table 1:** Characteristics of the study subjects.

	Case group (*n* = 48)	Control group (*n* = 90)	*P* value
**Men, %**	66.7	41.1	<0.01
**Age (yr), mean (SD)**	54.8 (10.4)	50.9 (10.3)	0.04
**Duration of stone work (yr), mean (SD)**	23.9 (15.2)	19.2 (13.7)	0.08
**BMI (SD)**	26.0 (3.4)	24.9 (4.3)	0.12
**FVC (% of predicted) (SD)**	85.3 (15.4)	94.7 (14.2)	<0.01
**FEV1 (% of predicted) (SD)**	87.1 (16.1)	95.4 (17.1)	<0.01
**FEV1/FVC (%) (SD)**	84.0 (6.8)	84.2 (5.7)	0.87
**FEF25-75 (% of predicted) (SD)**	83.3 (24.4)	86.7 (25.0)	0.44
**FeNO (ppb) (SD)**	27.0 (23.7)	30.2 (19.8)	0.39
**Cigarette smoking history**			
** Never smoked, %**^**a**^	50.0	73.0	
** Former smoker, %**	14.6	11.2	
** Current smoker, %**	35.4	15.7	
** Passive smoke exposure, %**^**b**^	47.9	41.1	
**Haemoglobin (g/dL), mean (SD)**	14.3 (1.4)	13.8 (1.5)	0.09
**Haematocrit (%), mean (SD)**	44.9 (4.1)	44.0 (4.0)	0.18
**White blood cell count (10**^**3**^**/μL), mean (SD)**	6.3 (1.3)	6.0 (1.7)	0.35
**SMRP (nM), mean (SD)**	0.7 (0.6)	0.68 (0.6)	0.89
**CEA (ng/mL), mean (SD)**	1.5 (1.7)	1.2 (1.3)	0.32
**Urinary 8-OhdG/creatinine (ng/mg), mean (SD)**	185.1 (393.2)	133.1 (65.2)	0.37
**Fibulin-3 (ng/mL), mean (SD)**	29.4 (35.4)	23.5 (30.4)	0.30

^a^ “Never smoked” indicates having smoked fewer than 20 packs of cigarettes in a lifetime or less than one cigarette per day for one year.

^b^ “Passive smoke exposure” indicates having been exposed to the smoke of others more than three times per week for more than six months.

## Discussion

Workers exposed to asbestos-contaminated minerals have an increased risk of pneumoconiosis. With improvements in data extraction by machine learning techniques that include more clinically important variables, the prediction models have high accuracy. To the best of our knowledge, this is the first study demonstrating that a combination of serum and urinary biomarkers can be used to predict pneumoconiosis.

Our results showed an increased SMRP level in workers exposed to nephrite. SMRP is a differentiation antigen present on normal mesothelial cells of the pleura, peritoneum, and pericardium. Elevated SMRP levels are observed primarily in patients exposed to asbestos, especially those who had MPM. Robinson et al. first reported that serum SMRP was elevated in patients with MPM [29]. A large-scale case-control study using a MESOMARK kit reported that SMRP could be used as a biomarker for MPM with a cut-off value of 1.5 nM [21]. SMRP might also be a biomarker of exposure to asbestos without MPM. A study that enrolled subjects with asbestos exposure due to industrial activity from two Italian regions (Tuscany and Liguria) showed a high incidence of MPM. The mean SMRP value was 0.7 nM in subjects with asbestosis and 0.75 nM in healthy asbestos-exposed controls [30]. Demir et al. investigated serum levels of SMRP among individuals who were environmentally exposed to asbestos through contaminated soil [31]. The mean SMRP level was 0.85 nM in the asbestos exposure group. Park et al. monitored 538 subjects with histories of asbestos exposure who made claims for compensation in Australia. The mean SMRP level in healthy subjects who were exposed to asbestos was 0.79 nM [32]. In an Italian study on asbestos-exposed subjects in dock/shipyards, the median SMRP value in healthy subjects was 0.4 nM [33]. Based on these studies, the level of SMRP in healthy asbestos-exposed subjects ranged from 0.4 to 0.85 nM. In healthy subjects without asbestos exposure, the mean SMRP level was 0.23 nM, as reported by Rodriguez Portal et al.[34]. In our study, the mean SMRP values were 0.84 nM (SD 0.52) in workers exposed to nephrite and 0.60 nM (SD 0.30) in workers exposed to other minerals, suggesting possible asbestos exposure in these study subjects, especially among workers who processed nephrite.

In this study, we found that the fibulin-3 level was higher in the case group than in the control group. Fibulin-3 is an extracellular protein that is mainly distributed in the eye and blood vessel walls [35]. Fibulin-3 is associated with cellular proliferation and malignant transformation. An animal study showed that exposure to fluoro-edenite a natural environmental contaminant of asbestiform fibres in Biancavilla, Italy, increased fibulin-3 overexpression in alveolar and bronchiolar epithelial wall cells and the pulmonary interstitium [36]. Recent epidemiological studies have demonstrated that fibulin-3, either in the blood or pleural effusion, is a potential diagnostic biomarker for MPM [35]. Kaya et al. measured serum fibulin-3 levels in 43 patients with malignant mesothelioma and 40 healthy controls. Using a cut-off value of 36.6 ng/mL, the AUROC was 0.976 [37]. Fibulin-3 might also be a biomarker of exposure. Pass et al. measured the fibulin-3 levels in three cohorts in North America. The plasma fibulin-3 level in MPM ranged from 66.4 to 112.9 ng/mL, which was higher than the fibulin-3 level in asbestos-exposed persons without cancer, which ranged from 13.9 to 24.3 ng/mL [17]. In our study, the mean value of fibulin-3 was higher in workers exposed to nephrite (35.4 ng/mL, SD 44.4) than in workers exposed to other minerals (24.1 ng/mL, SD 30.4) (*p*-value of one-tailed Wilcoxon rank sum test = 0.12). In this study, the case group had a higher proportion of current smokers. To prevent the influence of smoking, we further excluded active and former smokers and matched for age (± 5 years). The mean SMRP levels were 0.63 (SD 0.63) nM in the case group (*n* = 27) and 0.41 (SD 0.57) nM in the control group (*n* = 27) (*p* = 0.18). The mean fibulin-3 levels were 41.07 (SD 38.28) ng/mL in the case group and 16.67 (SD 22.06) ng/mL in the control group (*p* < 0.05). After eliminating the influence of smoking status, SMRP and fibulin-3 were still higher in the case group than in the control group. These results suggest that elevated SMRP and fibulin-3 are associated with asbestos exposure.

In this study, the CEA and 8-OHdG levels were higher in the case group than in the control group. CEA is a glycoprotein that is secreted into the luminal surface of the epithelia in the gastrointestinal tract. CEA levels can increase in colorectal cancer, gastric cancer, pancreatic cancer, lung cancer, ovary cancer, inflammatory diseases and with cigarette smoking [38–40]. 8-OHdG is a biomarker of oxidative stress and carcinogenesis [41]. Because CEA and 8-OHdG are not specific to dust exposure, we suggest that these two biomarkers should not be used alone to predict pneumoconiosis. Although the FVC and FEV1 levels were lower in the case group than in the control group, we observed that using the pulmonary function test alone had low predictive accuracy for pneumoconiosis because most of the cases were in the early stage of pneumoconiosis without clinical symptoms. Our study results suggest that the combination of lung function test, chest X-ray, and multiple biomarkers can improve the accuracy of screening for pneumoconiosis. The International Classification of High-resolution Computed Tomography for Occupational and Environmental Respiratory Diseases (ICORD) has been used for the screening and diagnosis of occupational disease [42]. We suggest that low-dose computed tomography can be used in high-risk workers who have been exposed to asbestos-contaminated minerals.

In the clinical setting, multiple tests are often used simultaneously. When multiple tests are used simultaneously to detect a specific disease, the individual is considered to have tested “positive” if he or she has a positive result on any one or more of the tests. The simultaneous testing will increase the net sensitivity [43]. For example, physicians in the emergency department will use several blood tests, urinary tests, and imaging studies to increase the overall sensitivity to diagnose a patient with fever of unknown origin. If we want to conduct an epidemiological study to evaluate the diagnostic accuracy using these tests, there will be a problem of multicollinearity for placing all of the tests (*X* variables) in a conventional multivariate logistic regression [44]. Machine learning has advantages in sophisticated algorithms that can handle non-linear data or problems of multicollinearity [45]. However, readers must know their application and limitations. While conventional statistics tend to emphasize inference, machine learning emphasizes prediction [46]. There may be a lack of well-understood relationships between independent and dependent variables. We suggest that it is important to include only important variables based on clinical knowledge and the biological and pathological mechanisms of the disease. The clinical diagnosis of pneumoconiosis may be difficult, but it is still essential for the further management of affected patients and for obtaining valid epidemiological data. Laboratory data and statistical modelling can be a valuable aid but can never replace a clinical workup.

## Conclusions

In this study, a health surveillance programme was conducted among stone workers of Hualien, Taiwan, some of whom processed nephrite, serpentine, and talc. We found that SMRP and fibulin-3 were increased in the subjects with pneumoconiosis, suggesting the possibility of exposure to asbestos. A combination of serum SMRP and fibulin-3, CEA, and urinary 8-OHdG can be used in health examinations to screen for pneumoconiosis in workers exposed to asbestos-contaminated minerals.
